# Learned optical flow for intra-operative tracking of the retinal fundus

**DOI:** 10.1007/s11548-020-02160-9

**Published:** 2020-04-22

**Authors:** Claudio S. Ravasio, Theodoros Pissas, Edward Bloch, Blanca Flores, Sepehr Jalali, Danail Stoyanov, Jorge M. Cardoso, Lyndon Da Cruz, Christos Bergeles

**Affiliations:** 1 University College London, London, UK; 2 King’s College London, London, UK; 3 Moorfields Eye Hospital NHS Foundation Trust, London, UK

**Keywords:** Synthetic data, Optical flow, Retinal tracking, Deep learning

## Abstract

**Purpose:**

Sustained delivery of regenerative retinal therapies by robotic systems requires intra-operative tracking of the retinal fundus. We propose a supervised deep convolutional neural network to densely predict semantic segmentation and optical flow of the retina as mutually supportive tasks, implicitly inpainting retinal flow information missing due to occlusion by surgical tools.

**Methods:**

As manual annotation of optical flow is infeasible, we propose a flexible algorithm for generation of large synthetic training datasets on the basis of given intra-operative retinal images. We evaluate optical flow estimation by tracking a grid and sparsely annotated ground truth points on a benchmark of challenging real intra-operative clips obtained from an extensive internally acquired dataset encompassing representative vitreoretinal surgical cases.

**Results:**

The U-Net-based network trained on the synthetic dataset is shown to generalise well to the benchmark of real surgical videos. When used to track retinal points of interest, our flow estimation outperforms variational baseline methods on clips containing tool motions which occlude the points of interest, as is routinely observed in intra-operatively recorded surgery videos.

**Conclusions:**

The results indicate that complex synthetic training datasets can be used to specifically guide optical flow estimation. Our proposed algorithm therefore lays the foundation for a robust system which can assist with intra-operative tracking of moving surgical targets even when occluded.

## Introduction

**Fig. 1:**
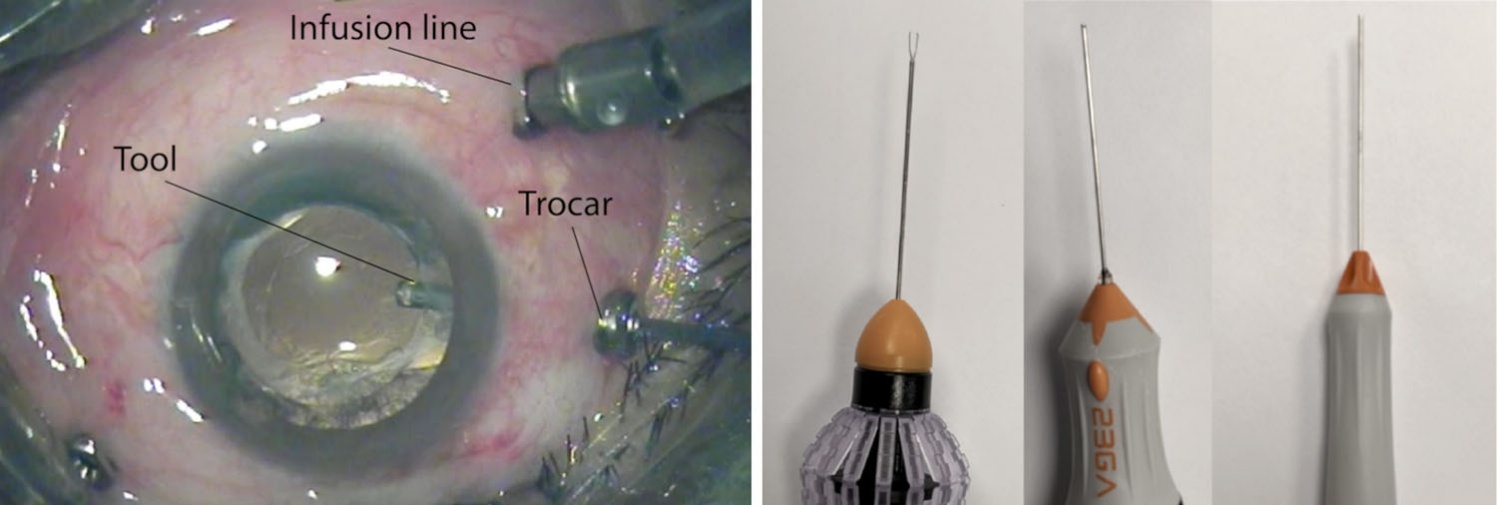
Left: illustration of a vitreoretinal surgical setup. Right: vitreoretinal surgical tools, from left to right: forceps, cutter, light pipe

Vitreoretinal surgery takes place within the gel-like vitreous humour of the eye, on top of the retinal surface, using a variety of tools of less than 0.7 mm diameter. The tools are inserted through trocar ports placed on the sclera, the white part of the eye (see Fig. 1). The surgeon operates without force perception, relying primarily on visual cues and feedback from stereo biomicroscopy providing a high-resolution view of the retinal surface. Recently, this en-face 2D view has been coupled with intra-operative optical coherence tomography (iOCT) as a complimentary imaging modality providing cross-sectional information of retinal layers.

**Fig. 2:**
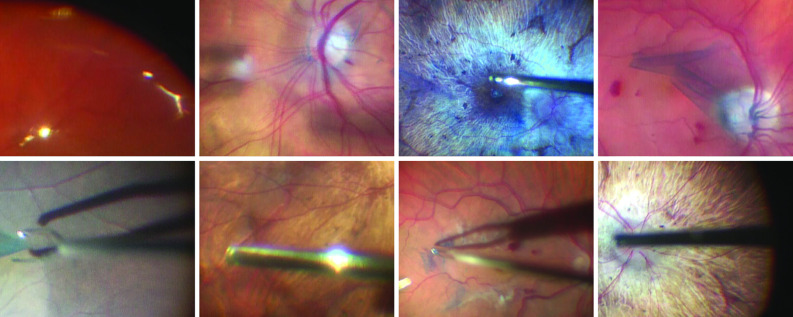
Challenges in intra-operative retinal surgery videos. Top row: reflections not located on the tool; double exposure from fast fundus motion; blue dye used to stain an epiretinal membrane (ERM); partially transparent peeled ERM fragments floating in front of the retinal fundus. Bottom row: tool hue and saturation blending with the background due to the retina being reflected on the metallic tool shaft; reflections/glare on the metallic tool; shadow cast by the tool on the retina; increasing width of the tool along its length due to its 3D pose

Common vitreoretinal surgical interventions like vitrectomy, epiretinal membrane peeling, laser delivery, etc. are routinely performed with very high success rates. However, upcoming vitreoretinal surgical procedures that promise to restore sight by delivering genes, stem cells [5], and small drug molecules to specific retinal layers require significantly more surgical precision. Preliminary clinical trials are encouraging, but for these therapies to be transformative, their sustained micro-precise delivery over several minutes to the retinal layers is required. The precision requirement will be met with novel robotic tools that are under development, while the promise of sustained delivery requires intra-operative image analysis to track the retinal fundus and support semi-automated therapy delivery. As due to patient breathing, cardiac pulsation, and surgical manipulation the retina deforms in a non-rigid fashion, robust frame-to-frame retinal fundus tracking in a highly challenging interventional environment, illustrated in Fig. 2, is required.

Our goal is to acquire dense optical flow predictions for the retinal fundus in real time, accounting for occlusions from surgical instruments, to enable tracking of any point on the retina throughout surgery.

*Prior work* Estimating optical flow to obtain motion cues from images is one of the oldest challenges in computer vision. Knowledge of optical flow in a temporal sequence of images can critically support high-level tasks such as image registration or deformation modelling in a variety of application domains [8].

Classical methods calculate optical flow as the solution to an optimisation problem. In its simplest form, the problem uses the optical flow constraint equation (OFCE) [17], linearised to work for small displacements. To overcome this limitation, multi-scale approaches were developed [25]. “Coarse-to-fine” adaptive algorithms refining the flow also proved successful [2]. Additional constraints to overcome the aperture problem [14] formulated as a prior term in the cost function [1] were introduced, either as a local parametric model or a global regularisation.

Occlusions, i.e. pixels visible in one frame that become hidden by objects in the next [8], have always posed challenges on optical flow estimation requiring occlusion detection and filling. The latter can be achieved with a diffusion-based method in the case of thin occlusions, or exemplar-based methods for larger areas [9], selecting motion candidates from surrounding pixels determined to belong to the same object. Earlier work in [17] noted that joint flow estimation and segmentation helped with extrapolating over larger occlusion areas. Flow estimation methods have also included learned components [3]. Recent advances in deep learning have spawned a wave of new algorithms, started off by the first end-to-end implementation, FlowNet, by Dosovitskiy et al. [6]. A wealth of different architectures have been proposed, e.g. using cascading networks [12], spatial image pyramids [19], and feature pyramids [24], as well as a number of multi-task networks such as joint depth and flow estimation, and unsupervised approaches.

Synthetic data has been used from the onset of optical flow estimation to overcome the intractability of manually annotating large datasets with dense motion vectors. Generated data complexity ranges from low-resolution simulations of a rotating patterned sphere [11] to animated scenes generated using modern 3D software. FlowNet, as trained by Dosovitskiy et al. [6], used the *Flying Chairs* dataset introduced in the same paper. Despite the domain gap between the synthetic dataset and real-world sequences, it was shown that FlowNet generalised well to real-world data such as the KITTI benchmark containing annotated road scenes. Mayer et al. [15] surveyed a number of synthetic datasets and concluded that data diversity helps, as does the simulation of distortions due to the camera.

Most previous work on tracking retinal fundus images based on classical computer vision approaches relies on detecting retinal vessels and registering vessel maps between successive frames [26]. Recently, Braun et al. [4] and follow-up work in Mukherjee et al. [16] succeeded in registering video frames to dynamically expanding and updating retinal vessel maps, allowing for camera motion estimation with respect to the retina where vessel features are visible. Richa et al. [21] on the other hand built a mosaic without explicitly extracting a vessel map using sum of conditional variance (SCV) tracking from image patches, a method nonetheless dependent on the presence of textured regions. Guerre et al. [10] created *Sliding Retinas*, comprising translations and rotations of retinal image patches. They found that while training FlowNet exclusively with *Sliding Retinas* did not yield useful results on this synthetic dataset, fine-tuning FlowNet after training with *Flying Chairs* first increased performance slightly compared to only using *Flying Chairs*. This was not quantitatively evaluated on real-world frame sequences. These reported methods cannot consistently track single points under occlusions.

## Materials and methods

We adopt a deep learning approach to both estimate optical flow and provide a segmentation containing information about whether a pixel belongs to the retinal fundus. The flow must ignore objects or effects not belonging to the retinal fundus so that points of interest on the retina, e.g. indicated for therapy delivery during a surgical procedure can be tracked continuously even when occluded.

**Fig. 3:**
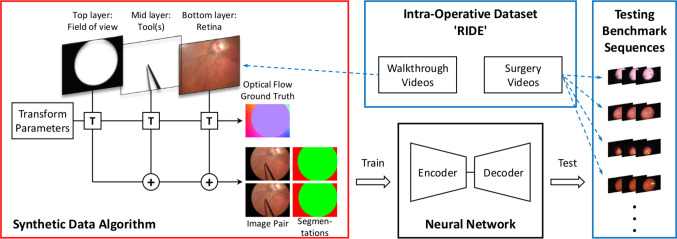
Overview of the algorithms presented in this paper; red frames refer to synthetic data, blue to real-world data. Left: the synthetic data algorithm, with the layers transformed [**T**] and combined (**+**) to result in a pair of images and corresponding segmentations. The ground truth optical flow results from the known transformations applied to the layers. The underlying retinal fundus layers are drawn from the RIDE walk-through videos (“Synthetic dataset generation” section). Top middle: the intra-operative dataset RIDE contains data recorded in the operating theatre—walk-through videos, videos of surgical procedures (“Intra-operative dataset RIDE” section). Bottom middle: the neural network, trained on the synthetic data, tested on the benchmark sequences (“Neural network architecture” section). Right: test benchmark of frames extracted from surgical procedure videos in RIDE (“Testing” section)

Learning to estimate optical flow in a supervised manner requires a large amount of ground truth optical flow training data. However, manually annotating image sequences is infeasible, especially in a challenging domain such as surgical data. We overcome this by generating synthetic datasets simulating intra-operative video frames. These datasets contain image pairs created by applying known transformations to composite images created from real-world data, which allows for the knowledge of the optical flow ground truth. Further, the datasets contain corresponding pairs of segmentation masks marking whether a pixel is inside the microscope field of view, and therefore tracked as part of the retina, or not. By replicating the phenomena observed in the surgical videos (see examples in Fig. 2) on the images but suppressing them in the ground truth flow, the network learns to ignore them when estimating the flow between frames of intra-operative videos, implicitly inferring the optical flow of occluded from non-occluded retinal regions.

While a random validation set is used to evaluate the network’s performance on the *synthetic* data, we only report the results of testing on *real-world* data.

### Intra-operative dataset RIDE

The real-world data used in this report originate from an internal dataset called “RIDE” (Retinal Image Database Elements), which consists of retinal image and video data from 66 patients acquired during a variety of vitreoretinal surgeries at Moorfields Eye Hospital, London, UK. All intra-operative biomicroscopy sequences were captured through a ZEISS OPMI Lumera 700 microscope using its integrated camera at a resolution of ${1920} \times {1080}\,\hbox {px}$ and at 25 frames per second. The videos acquired for each patient are split into two categories:

1. “Walk-through” videos, recorded in the usual intra-operative set-up, with a light pipe inserted into the vitreous but before the surgical procedure has started. The surgeon pivots the eyeball to visualise different parts of the retina, and the lighting is varied by moving the light pipe. These data aim to capture the distinct visual appearance of the retina across patients and are used to construct the synthetic datasets and to *train* the neural network.
2. Surgical procedure videos showing unadulterated real-world situations as recorded during a variety of interventions such as pars plana vitrectomy (PPV), inverted internal limiting membrane (ILM) flap technique, epiretinal membrane (ERM) peeling, and gene therapy injections. These data represent what the surgeon sees through the microscope and are used to *test* the neural network.

Despite using simple walk-through video frames as the basis of training the optical flow estimation network, the process we establish for data generation allows the generalisation of the network to *unseen* and *complex* interventional videos.

### Synthetic dataset generation

Every image seen through the microscope comprises the retinal fundus as a bottom, surgical tools as a middle, and the border of the microscope field of view as a top layer (see Fig. 3). The algorithm developed to create synthetic image pairs is based on this arrangement. For each image pair to be synthesised, instances of the three layers are created, each including an opacity channel encoding shape information, e.g. of the tools. A sequential combination of transformations on the layers results in the per-layer optical flow ground truth. Using the opacity channels as masks, the layers are merged resulting in an image pair, the completed optical flow ground truth, and segmentation ground truths. Image noise and compression artefacts are added to the image pair to mimic the quality of the real-world videos.

The most prominent of the phenomena observed in the intra-operative dataset RIDE have been implemented as transformation functions that can be applied to any of the image layers described above. Explicitly defining these transformations for each record in datasets of thousands of synthetic image pairs is not realistic. Therefore, we generate random transformations by sampling parameters from uniform distributions based on observations on interventional videos, e.g. with regard to maximum rotations to be expected between two video frames.

*Bottom layer—the retinal layer* Clips of 10 s duration showing the maximum area of retinal fundus are extracted from each walk-through video. Given the camera frame rate of 25 frames per second, each clip contains, per patient, 250 3-channel colour images at a resolution of $1920 \times 1080\,\hbox {px}$. An area containing only retinal fundus is identified by finding the largest possible rectangle that fits into the image region with a brightness below an adaptive threshold. Median filtering ($3\,\hbox {px}$ kernel size) was used to reduce compression artefacts and image noise while preserving small-scale features such as thin vessels.

*Middle layer—the tool layer* All tools shown in the synthetic datasets—light pipe, cutter, and forceps—are based on one template each, manually segmented from single images of these tools captured against a white background (see Fig. 1). A number of transformations are applied to the templates:

- Template scaling, ranging from 0.8 to 1.5 to match tool sizes in real images.
- Simulation of a 3D tool pose by stretching the tool shaft width as it moves away from the centre of the image, ranging from 1.5 to 3.
- Template rotation between $-\,80^{\circ }$ and $80^{\circ }$ off the horizontal axis.
- Placement of tool tip on a point in the image between 0.2 and 0.8 of the image height, and 0.15 and 0.5 of the image width if the tool is a light pipe, or between 0.4 and 0.85 of the image width for all other tools.
- Variable Gaussian blurring matching observed real tool appearance (Fig. 2), kernel size ranging from 3 to $7\,\hbox {px}$.
- Conversion into HSV colourspace: the hue and saturation are adjusted to match the average colour of the retinal fundus image the tool will be overlaid on, while the value is randomly sampled between black and the value of the retinal fundus. This transformation accounts for the retina being reflected on the metallic tool, as well as the varying illumination of tools in the videos.
- Creation of a shadow as the projection of the tool onto the retina. The offset, angle, and transparency are sampled from uniform distributions between 0 and $70\,\hbox {px}$, $-\,45^{\circ }$ and $45^{\circ }$, and 0 and 0.5, respectively.
- Positioning of glaring along the centreline of the tool. This is a common phenomenon that is difficult to model realistically due to the complexity of the interaction between light and the reflective metal tool shafts. We approximate it by a number of connected, white, faded ovals with a yellow and blue-coloured crest added to the outline. The number of ovals and their radius are sampled from a uniform distribution between 1 and 5 and $2\,\hbox {px}$ and $12\,\hbox {px}$, respectively, while transparency of the coloured crest is chosen between 0.1 and 0.6.

*Top layer—the “field of view” layer* The third and last layer simulates the effect of the microscope, limiting the field of view to a circular area with blurred edges. All areas exterior to the field of view are black. The radius of this circle is sampled from a uniform distribution between 0.4 and 0.8 of the image height, and the centre position lies between 0.4 and 0.6 of the image height and width.

**Fig. 4:**
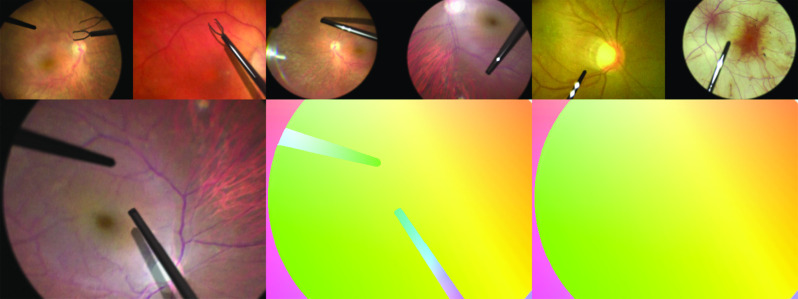
Top row: sample synthetic images showcasing representative retinal appearances, tools, reflections, motion blur, and artificial noise. Bottom row: a sample image from a synthetic data image pair, the corresponding full ground truth flow, and the simplified ground truth flow used for suppressing the occlusions generated by the tools. Optical flow vector direction is encoded in the hue, magnitude in the saturation (as in [6])

*Layer transformations* Each of the layers is then transformed in different ways to arrive at two distinct images, the original image and the transformed image, with a known optical flow between them. Flow attributable to tool layers is removed from this ground truth (see Fig. 4) to obtain training data that will lead the network to learn to ignore tools and related effects such as shadows in the test surgical intervention videos. Transformations are applied to the layers by type:

- *Transformations of the layer itself* Local distortions simulating bubbling due to an injection; geometric transformations such as translation, rotation, and scaling, simulating the appearance changes observed in RIDE due to movements of the microscope, tools, or the eye itself. The latter transformations have a modifier that allows a “double exposure” effect to be simulated. Such effects appear in RIDE videos when sudden eye motion takes place.
- *Lens effect* Pincushion distortion, simulating the distortion occurring towards the edges of the microscope field of view.
- *Transformations without flow or segmentation ground truth impact* local or global intensity and contrast changes, blurring, tool shadows, glare.

*dataset parameters* The main dataset comprises 16 subsets of 2000 records each for a total of 32,000 image pairs, the same order of magnitude as the Flying Chairs dataset Dosovitskiy et al. [6]. Resolution was chosen as ${512} \times {384}\,\hbox {px}$, balancing the need to retain the details of important retinal features with the need to reduce the computational overhead of creating and training on larger-resolution data.

The first five subsets contain just the retinal layer transformed as follows: translation up to $10\,\hbox {px}$, rotation $-\,5^{\circ }$ to $5^{\circ }$, scaling 0.9–1.1, pincushion distortion maximum 10–$50\,\hbox {px}$, and “bubbling” centred between 0.2 and 0.8 of the image height and width, with a radius 0.15*h* to 0.3*h*, *h* being the image height. The next five subsets contain a retinal and a field of view layer, with the following permutations of transformations (same values as before): translation, rotation, scaling, local distortion, rotation, and scaling. The final six subsets contain a retinal layer, one or two tool layers, and a field of view layer, each with translation, rotation, and scaling. To provide the network with data of varying complexity and appearance, every layer in every subset has 10% chance of Gaussian blur with a kernel size up to $5\,\hbox {px}$; 25% chance of global brightness changes up to 15; 10% chance of local brightness changes up to 40 (patch radius 0.1*h*–0.3*h*). These hyperparameters were chosen based on a visual inspection of our intra-operative dataset RIDE and in accordance with guidance from our clinical collaborators. Clinicians also visually evaluated representative examples of the generated sequences for their realism.

Two additional datasets were created with the same parameters, but experiencing fewer transformations: “dataset-nl” contains no tool shadows or reflections, while the tool hue and saturation are not adjusted to match the retinal fundus. “Dataset-nl-nb” is further “handicapped” by not including any brightness changes.

### Neural network architecture

The network employs the well-performing, fully convolutional “FlowNetSimple” architecture [6] with a classic encoder–decoder structure and skip connections. Two ways for segmentation prediction were implemented: once as a separate decoder branch following the design of the fully convolutional network by Shelhamer et al. [22] (called “FNS-branch” in this paper), and once as additional channels of the prediction tensor, which is then split into the optical flow and the semantic segmentations (called “FNS-comb”). A leaky rectified linear unit (l-ReLU) with a slope of 0.01 was chosen as the standard activation function [23]. Further modifying FlowNet, loss is calculated as an average across scales. This has been shown to work well in applications such as deblurring [18] and unsupervised optical flow prediction [20], and mimics successful long-established variational methods [25].

*Cost function* The cost function consists of four loss terms *L* with weightings $\lambda$: the flow loss, a regularisation loss intended to reduce overfitting, the segmentation loss, and a total variation loss that promotes a smooth flow field:

1 $$\begin{aligned} \mathrm{Cost}&= \lambda _{\mathrm{flow}} \frac{1}{n_\mathrm{f}} \sum _{s,\; x,\; y}\left\Vert f_{s,\; x,\; y}^{\mathrm{pred}} - f_{s,\; x,\; y}^{gt} \right\Vert \nonumber \\&\quad +\,\lambda _{\mathrm{reg}} \frac{1}{2} \sum _{l,\; i,\; j} w_{l,\; i,\; j}^2 \nonumber \\&\quad +\,\lambda _{\mathrm{seg}} \sum _{s,\; x,\; y,\; i} -p_{s,\; x,\; y,\; i}^{gt}\; \mathrm{log} (p_{s,\; x,\; y,\; i}^{\mathrm{pred}}) \nonumber \\&\quad +\,\lambda _{\mathrm{var}} \sum _{x,\; y} m_{x,\; y} \left( \frac{\partial f_{x,\; y}^{\mathrm{pred}}}{\partial x} + \frac{\partial f_{x,\; y}^{\mathrm{pred}}}{\partial y}\right) \end{aligned}$$

where the flow weighting $\lambda _{\mathrm{flow}}$ is always 1, $n_\mathrm{f}$ is the number of predicted flow vectors *f*; $p_i$ is the probability of segmentation class *i*; superscripts pred and *gt* stand for “prediction” and “ground truth”, subscript *s* for scale, $x,\;y$ for spatial coordinates, *l* for layer, *j* for spatial coordinates in feature space; *m* is a Boolean array preventing the gradient at field of view borders from being smoothed.

*Training parameters* Each subset of the synthetic dataset is randomly split into 95% training, 5% validation, used exclusively to verify that the network is indeed learning. Recall that final testing is performed in real-world vitreoretinal surgical intervention videos and not the synthetic data. Following the original FlowNet paper, the Adam optimiser with default parameters ($\beta _1 = 0.9, \beta _2 = 0.999$) was used. From experimentation on validation data, a mini-batch size of 10 was chosen, and the learning rate decayed exponentially from an initial value of $10^{-4}$ with a decay rate of 0.95 and a decay step value of $10^{4}$. The loss term weightings $\lambda _{\mathrm{seg}}$, $\lambda _{\mathrm{reg}}$, $\lambda _{\mathrm{var}}$ were set as $10^{-3}$, $10^{-7}$, and $10^{-6}$, respectively. Training was run for 100 epochs as a standard to guarantee convergence on real data without having to test the benchmark repeatedly. Training and testing was run on a server with an NVIDIA Quadro P6000 GPU, an Intel i7-6900K 16-core CPU, and $64\,\hbox {GB}$ RAM.

### Testing

We curated a challenging benchmark comprising unseen real-world clips from the collected surgical procedure videos for testing. Our evaluation only includes points on the retina and ignores motion estimations for areas outside the field of view.

*Benchmark “BM”* 32 clips, 8 s long for a total of 201 frames each, originating from videos of surgical procedures from the RIDE dataset. This benchmark cannot be selected randomly, as that would include too many sequences without tools or with very little movement. The following selection criteria were applied:

- Continuous large-scale (rotation $>5^{\circ }$, translation $> 10\,\hbox {px}$, scaling $>10$%) retinal motion in 10 $+$ frames without continuous blur over the same period.
- Tools moving across part of the visible retinal fundus.
- Presence of at least some of the phenomena from the top two rows in Fig. 2.

The result is a collection of challenging clips representative of surgical procedures. Resolution is reduced to $512 \times 384\,\hbox {px}$ to match the training data scale.

*Point tracking* In each sequence, four ground truth points located on strong features such as vessel bifurcations were annotated every ten frames. For the same frames, a binary map indicating whether a pixel is located inside the field of view of the microscope was created. The ground truth points are tracked throughout each sequence, forwards and backwards. The mean L2 distance from the ground truth at the 201-st (last) frame, i.e. the end-point error (EPE), is called *long sequence EPE (l-EPE)*. The same is done for each 11-frames-long sequence fragment with ground truth annotation at the start and the end. The overall mean L2 distance from the ground truth being called *short sequence EPE (s-EPE)*—this is our main metric and the closest proxy for frame-on-frame flow estimation errors. We report the s-EPE and l-EPE mean and standard deviation across the benchmark.

*Grid tracking* Sequence EPEs are limited by the both temporally and spatially sparsely available ground truths. To get a dense error estimation despite this, we overlay a grid on the first frame of each sequence, using the field of view segmentation to limit it to the area recognised as retinal fundus. The grid vertices are then tracked in a “loop” throughout the benchmark sequence, on odd frames from 1 to 201, then back to 1 on even frames. This ensures the algorithm cannot achieve a zero tracking error with a random but invertible prediction. An ideal tracking system would restore the grid vertices back to the original position, the initial and final grid being congruent. L2 distances of end to start vertex positions, termed *grid EPE*, represent a quality measure for tracking of the entire retina.

A “grid” size of a single pixel was chosen, implying that $10^{6}$ pixels are tracked throughout the benchmark sequence. This choice maximises how representative of the overall tracking quality the statistics of the grid errors are. We report the average grid EPE and average grid EPE standard deviation over the benchmarks.

Since intuitive interpretation of optical flow visualisation is challenging, grid tracking additionally allows for a qualitative evaluation of the output. The motions and deformations of a superimposed grid are easily appreciated, while the underlying image is clearly visible as well.

*Benchmark without occlusions “BM-no-occ”* In addition to “BM”, a different set of 10 8 s clips was also compiled. The ground truth points in “BM-no-occ” are never occluded by tools, glare, or similar effects, and were specifically chosen to allow a more direct comparison with the variational methods (see “Experiments and results” section).

## Experiments and results

Two network designs *FNS-branch-ms-seg* and *FNS-comb-ms-seg* as well as *FNS-comb* variants without segmentations and/or without multi-scale loss were tested. The suffix “ms” refers to the use of multi-scale loss, the suffix “seg” to the inclusion of a segmentation prediction. The validation EPE on the synthetic data converges to a value of about $0.2\,\hbox {px}$ for *FNS-comb-ms-seg*, but rather than evaluating performance on the synthetic data, we are primarily interested in tracking unseen surgical procedure clips. To quantify the effect of dataset size and fidelity, *FNS-comb-ms-seg* was also tested with the width reduced from 38 m learned parameters to 75% and 50%, with the data split adjusted from 95% for training to 65% and 35%, and on the two simplified datasets “dataset-nl” and “dataset-nl-nb”.

Two variational methods are included as a baseline: the Farnebäck method [7] included in OpenCV (termed *FNB*), and the coarse-to-fine SIFT flow [13] (termed *C2F*) implemented in C++ by its author^1^ and provided with a Python wrapper.^2^ All methods that do not have segmentations were provided with a ground truth segmentation every 10th frame (see “Testing” section) to constrain tracking to the retina.

All networks run at over 50 Hz for input images at $512 \times 384\,\hbox {px}$, which makes them suitable for real-time applications in the operating theatre even at a higher resolution. The variational methods are significantly slower: FNB at 8 Hz, C2F at less than 0.2 Hz, partially due to being implemented on CPU rather than GPU.

**Table 1:** Experimental results

	BM-no-occ	Evaluation benchmark BM		
	s-EPE	s-EPE	l-EPE	Grid EPE
Baseline				
FNB	**2**.**0** (4.4)	3.8 (7.2)	24.1 (30.7)	14.0 (17.4)
C2F	2.3 (4.1)	4.2 (9.5)	26.4 (33.4)	13.3 (16.3)
Network type				
FNS-branch-ms-seg	2.1 (2.4)	2.7 (2.7)	16.0 (10.8)	18.8 (9.4)
**FNS-comb-ms-seg**	**2.0 (2.3)**	**2.6 (2.6)**	**14.7 (7.9)**	**15.8 (7.9)**
FNS-comb-ms	2.3 (2.4)	2.9 (2.7)	19.5 (13.3)	21.9 (11.0)
FNS-comb-seg	2.4 (2.5)	3.0 (3.0)	19.7 (13.2)	23.5 (10.1)
FNS-comb	2.8 (2.8)	3.3 (3.0)	25.8 (14.7)	29.2 (13.2)
Network ablation				
75% width	2.6 (2.7)	2.9 (2.7)	23.5 (17.3)	29.3 (13.4)
50% width	2.7 (2.9)	3.3 (3.2)	25.1 (17.5)	26.4 (12.8)
Dataset ablation				
65% for training	2.6 (2.7)	3.0 (2.7)	21.3 (12.4)	26.1 (11.5)
35% for training	2.7 (2.7)	3.4 (3.3)	30.0 (20.8)	28.6 (11.6)
Dataset versions				
Dataset-nl	2.3 (2.4)	3.1 (3.0)	23.2 (13.3)	25.6 (10.7)
Dataset-nl-nb	2.9 (2.8)	4.3 (7.0)	29.6 (21.9)	29.8 (12.7)

Bold values denotes best results in each category (or equal best results if there is more than one)

All errors in pixels, reported as “mean (standard deviation)”

**Fig. 5:**
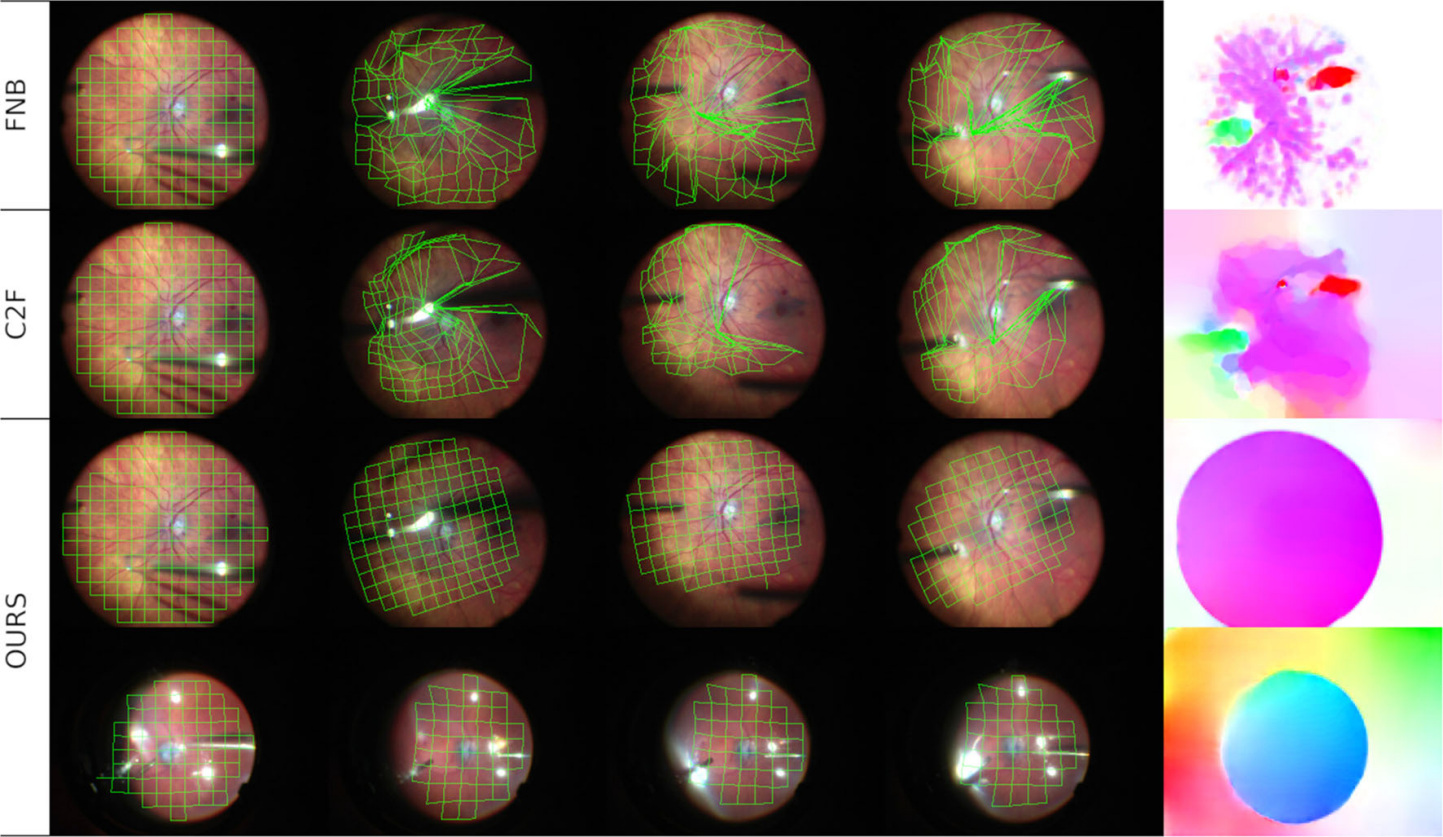
Illustration of grid tracking and flow field. 1st Row: benchmark sequence $\#4$, evaluated with *FNB*. 2nd Row: benchmark sequence $\#4$, evaluated with *C2F*. 3rd Row: benchmark sequence $\#4$, evaluated with *FNS-comb-ms-seg* (ours). 4th Row: benchmark sequence $\#3$, evaluated with *FNS-comb-ms-seg*. Videos accessible at bit.ly/2ObYdHi

All experimental results are collated in Table 1. The different metrics show high consistency: higher short (11 frames) sequence EPEs (s-EPE) are matched by higher long (201 frames) sequence EPEs (l-EPE), due to higher frame-to-frame tracking errors compounding to a much larger overall error on longer sequences. The mean grid error, not compared with a ground truth, is highly correlated with the ground-truth-based s-EPE, validating the grid EPE as an evaluation method. The one outlier is the grid errors of the variational baseline methods. This is explained by Fig. 5, which shows a comparison of tracking quality. *FNB* tracks strong features such as vessels and the tool very well, but many points in-between have an estimated flow of zero: some grid vertices move very little over the course of the clip, resulting in a low grid error mean but high standard deviation. *C2F* is more successful at interpreting the retinal fundus as an object moving “as one”, but it cannot ignore occlusions arising from surgical tools; the flow prediction at such object borders is imprecise. As a consequence, the grid moves with the retinal fundus to some degree, but is deformed in many locations. *FNS-comb-ms-seg*, by contrast, is able to track the grid while ignoring tools, shadows, and reflections.

*FNS-comb-ms-seg* and *FNS-branch-ms-seg* perform best overall, with “BM” s-EPE values of 2.6 px and 2.7 px, respectively. Network versions without simultaneous segmentation prediction or multi-scale loss perform consistently worse. All variants outperform the variational baseline methods, with corresponding values of 3.8 px and 4.2 px. Tracking points not experiencing any occlusions in “BM-no-occ” is less challenging, and all methods evaluate to significantly lower s-EPE values. *FNS-comb-ms-seg* has the same mean as FNB, but achieves a lower standard deviation. This can be observed with all deep learning variants: even though average performance might degrade due to less training data or simpler datasets, the standard deviation remains lower than for the variational methods, indicating an ability to track the retina as one object.

Training a thinner network reduces tracking performance on “BM” to a mean s-EPE of $2.9\,\hbox {px}$ and $3.3\,\hbox {px}$, which is consistent with a reduction of the representational capacity of the network. Training on a fraction of the synthetic data similarly increases s-EPE means to $3.0\,\hbox {px}$ and $3.4\,\hbox {px}$: this suggests enriching the training dataset could lead to performance improvements. We also note that training on simplified synthetic data degrades performance to $3.1\,\hbox {px}$ for “dataset-nl” and $4.3\,\hbox {px}$ for “dataset-nl-nb”. This is a strong argument in favour of improvements of the simulation of phenomena observed in RIDE videos (see Fig. 2).

## Conclusions and outlook

This paper presented a method to track points on challenging intra-operative retinal fundus videos through optical flow estimation. We demonstrated that robust optical flow estimation can be achieved by learning from complex synthetic data. Optical flow is implicitly inpainted underneath surgical tools, enabling the tracking of the retinal fundus even during occlusions in real surgical videos. Finally, our results corroborate that optical flow prediction and semantic segmentation, in this instance of the field of view, are mutually supportive tasks. This paves the way for further investigation of tool segmentation as a secondary task. In the future work, we will also investigate hyperparameter optimisation to increase performance.

## Footnotes

1 https://people.csail.mit.edu/celiu/OpticalFlow.

2 https://github.com/pathak22/pyflow.
